# Sarcopenia Adversely Affects Outcomes following Cardiac Surgery: A Systematic Review and Meta-Analysis

**DOI:** 10.3390/jcm12175573

**Published:** 2023-08-26

**Authors:** Ali Ansaripour, Arian Arjomandi Rad, Marinos Koulouroudias, Dimitrios Angouras, Thanos Athanasiou, Antonios Kourliouros

**Affiliations:** 1 Department of Cardiothoracic Surgery, John Radcliffe Hospital, Oxford University Hospitals NHS Foundation Trust, Oxford OX3 9DU, UK; ali.ansaripour@gtc.ox.ac.uk; 2 Medical Sciences Division, University of Oxford, Oxford OX1 3AZ, UK; 3 Department of Cardiac Surgery, Nottingham University Hospitals NHS Trust, Nottingham NG5 1PB, UK; 4 Department of Cardiac Surgery, Attikon University Hospital, National and Kapodistrian University of Athens, 10679 Athens, Greece; 5 Department of Surgery and Cancer, Imperial College London, London SW7 2BX, UK

**Keywords:** sarcopenia, cardiac surgery, frailty

## Abstract

Background: Sarcopenia is a degenerative condition characterised by the loss of skeletal muscle mass and strength. Its impact on cardiac surgery outcomes remains poorly investigated. This meta-analysis aims to provide a comprehensive synthesis of the available evidence to determine the effect of sarcopenia on cardiac surgery outcomes. Methods: A systematic review and meta-analysis followed PRISMA guidelines from inception to April 2023 in EMBASE, MEDLINE, Cochrane database, and Google Scholar. Twelve studies involving 2717 patients undergoing cardiac surgery were included. Primary outcomes were early and late mortality; secondary outcomes included surgical time, infection rates, and functional outcomes. Statistical analyses were performed using appropriate methods. Results: Sarcopenic patients (906 patients) had a significantly higher risk of early mortality (OR: 2.40, 95% CI: 1.44 to 3.99, *p* = 0.0007) and late mortality (OR: 2.65, 95% CI: 1.57 to 4.48, *p* = 0.0003) compared to non-sarcopenic patients (1811 patients). There were no significant differences in overall surgical time or infection rates. However, sarcopenic patients had longer ICU stays, higher rates of renal dialysis, care home discharge, and longer intubation times. Conclusion: Sarcopenia significantly increases the risk of early and late mortality following cardiac surgery, and sarcopenic patients also experience poorer functional outcomes.

## 1. Introduction

Sarcopenia, a degenerative condition characterised by the progressive and generalised loss of skeletal muscle mass and strength, is increasingly recognised as a significant health concern [1]. It is commonly associated with aging, and its prevalence is particularly noticeable in the elderly. Sarcopenia has been linked to a broad range of adverse health outcomes, including impaired physical function, increased risk of falls, prolonged recovery periods, and higher mortality rates [2]. In the context of cardiac surgery, sarcopenia’s implications are even more profound. As surgical techniques and medical management continue to advance, a growing number of older adults are becoming candidates for cardiac surgery. However, age-related conditions like sarcopenia pose unique challenges in this demographic, often complicating their post-operative recovery and overall prognosis [3,4].

Despite the mounting evidence demonstrating the impact of sarcopenia on surgical outcomes, it remains a poorly recognised and underdiagnosed entity in cardiac surgery. This discrepancy, in part, could be attributed to the complexity surrounding the diagnostic criteria of sarcopenia. There is no universal consensus on the definition of sarcopenia, with multiple working groups suggesting different guidelines. Notably, the European Working Group on Sarcopenia in Older People (EWGSOP) [5], the Asian Working Group for Sarcopenia (AWGS) [6], and the Foundation for the National Institutes of Health [7] each provide their unique sets of criteria, differing in their approach to quantifying muscle mass, muscle strength, and physical performance. This variability in diagnostic criteria has led to discrepancies in reported prevalence rates and has created challenges in comparing results across studies. In the past decade, the body of research investigating the impact of sarcopenia on cardiac surgery has grown substantially. Still, the findings have been somewhat inconsistent due to variations in study designs, the heterogeneity in patient populations, and the divergence in defining sarcopenia.

While obesity has been studied extensively and identified as a risk factor for poor outcomes in cardiac surgery [8,9], body mass index (BMI) is an inaccurate indicator of the relationship between adiposity and muscle mass [10]. Indeed, a sarcopenic patient can exhibit a normal or even elevated BMI that would qualify them as having sarcopenic obesity, a condition disregarded in previous studies only concentrating on BMI on cardiac surgical outcomes [11].

Through this meta-analysis, we aim to provide a more robust estimate of the actual effect size of sarcopenia on cardiac surgery outcomes. By highlighting the burden of sarcopenia in cardiac surgery, we hope to promote the development of targeted interventions and the incorporation of sarcopenia screening into preoperative evaluations. Furthermore, this analysis could provide insights into future research areas, particularly interventional studies aiming to mitigate the impact of sarcopenia on cardiac surgery outcomes.

## 2. Materials and Methods

### 2.1. Literature Search Strategy

**Figure 1:**
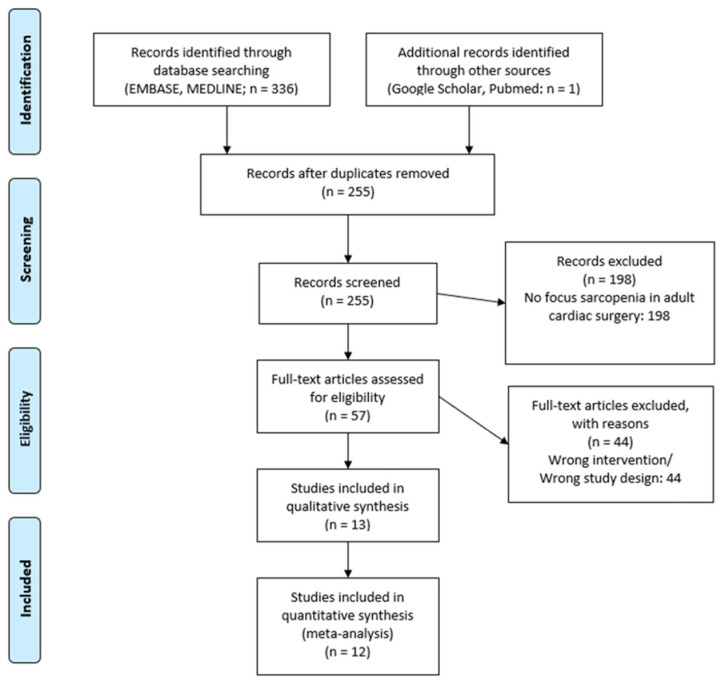
PRISMA Flow Chart.

A systematic review and meta-analysis was conducted in accordance with the Cochrane Collaboration published guidelines and the Preferred Reporting Items for Systematic Reviews and Meta-Analyses (PRISMA) statement. A literature search was conducted of EMBASE, MEDLINE, Cochrane, PubMed, and Google Scholar from inception to April 2023 (Figure 1). The search terms used were: (“Sarcopenia” OR “Muscle Wasting” OR “Muscle Mass” or “Muscle Atrophy”) AND (“Cardiac Surgery” OR “Cardio-thoracic Surgery” OR “Heart Surgery” or “Surgical Aortic Valve Replacement” or “Coronary Artery Bypass Grafting” or “aortic surgery” or “mitral valve surgery”). Further articles were identified using the ‘related articles’ function on MEDLINE and a manual search of the references lists of articles found through the original search. The only limits used were the English language and the mentioned time frame. Patient consent and institutional review board approval were unnecessary in this study as no patients were recruited.

### 2.2. Study Inclusion and Exclusion Criteria

All original comparative articles of patients with or without sarcopenia undergoing adult cardiac surgery and reporting on mortality and morbidity outcomes were included. Studies were excluded from the review if: (1) inconsistencies in the data precluded valid extraction; (2) the study was performed in an animal model; (3) studies did not have a comparison group; or (4) the size of the study population was small (<10 patients). Case reports, reviews, abstracts from meetings and preclinical studies were excluded. Using the above criteria, two reviewers (AA and A.AR.) independently selected articles for further assessment after the title and abstract review. A third independent reviewer (T.A.) resolved disagreements between the two reviewers. Potentially eligible studies were then retrieved for full-text assessment.

### 2.3. Data Extraction and Critical Appraisal

All full texts of retrieved articles were read and reviewed by two authors (A.A. and A.AR.), and the inclusion or exclusion of studies was decided unanimously. When there was disagreement, a third reviewer (T.A.) made the final decision. Using a pre-established protocol, the following data were extracted: first author, study type and characteristics, number of patients, population demographics, stroke rate, overall stroke rate, major bleeding, cardiopulmonary bypass (CBP) time, hospital length of stay, kidney dysfunction, early mortality, and overall mortality. For this review, a data extraction sheet was developed and pilot-tested on three randomly selected included studies, whereupon the sheet was refined accordingly. Data extraction was performed by two review authors (A.A and A.AR.). A third author (T.A.) validated the correctness of the tabulated data. Potential inter-reviewer disagreements were resolved by consensus. Primary outcomes were early/overall mortality. Secondary outcomes were hospital length of stay (LOS), intensive care unit (ICU) LOS, cross-clamp (CC) time, CBP time, overall surgery time, postoperative arrhythmias, sternal wound infection, stroke, kidney failure, discharge to care home, and intubation time.

### 2.4. Data Analysis

Odds ratios (OR) with 95% confidence interval (CI) and *p*-values were calculated for each categorical clinical outcome. Additionally, we utilised the Mean Difference (MD) as a statistical analysis method to analyze continuous data in our meta-analysis. MD enabled us to quantify the absolute difference in means between two groups, providing insights into the magnitude of effect size. Forest plots were created to represent the clinical outcomes. Chi-squared and I2 tests were executed for the assessment of statistical heterogeneity. Using a Mantel-Haenszel random-effects model, the ORs were combined across the studies. Funnel plots were constructed to assess publication bias. All analyses were completed through the “metafor” package in R Statistical Software (version 4.0.2) (Foundation for Statistical Computing, Vienna, Austria). A two-tailed *p*-value < 0.05 was considered statistically significant.

### 2.5. Sensitivity Analysis

The influence of a single study on the overall effect of sarcopenic versus non-sarcopenic patients undergoing adult cardiac surgery on the primary outcome was assessed by sequentially removing one study (the “leave-one-out” method). This sensitivity analysis was carried out to test the consistency of results to investigate if individual studies had an excessive impact on the analysis across all outcomes.

## 3. Results

### 3.1. Description of Studies

The literature search identified 336 articles. Of these, 57 relevant articles were read in full and assessed according to our inclusion and exclusion criteria. Following critical appraisal, a total of 12 studies [12,13,14,15,16,17,18,19,20,21,22,23] incorporating a total of 2717 patients were included. The studies described outcomes of sarcopenic (906) versus non-sarcopenic (1811) patients undergoing adult cardiac surgery. Figure 1 illustrates the study selection process.

### 3.2. Baseline Characteristics

There were 2717 patients included in this meta-analysis, of which there are 906 sarcopenic and 1811 non-sarcopenic patients. The data on baseline characteristics can be found in Table 1, Supplementary Figure S1. The mean age of the patients in the sarcopenic and non-sarcopenic cohorts was 70.57 and 65.36 years, respectively. In terms of BMI, the mean for the sarcopenic group was 22.21, while for the non-sarcopenic group, it was 23.73. The mean Left Ventricular Ejection Fraction (LVEF) percentages were 57.32% and 57.86% for the sarcopenic and non-sarcopenic groups, respectively. Lastly, the mean psoas muscle area index for the sarcopenic group was 478.2 mm²/m²; for the non-sarcopenic group, it was 819.6 mm²/m². The mean percentages of sarcopenic and non-sarcopenic patients with diabetes were 41.60% and 34.41%, respectively. Similarly, the mean percentages of sarcopenic and non-sarcopenic patients with hypertension were 70.39% and 73.20%, respectively. In the case of chronic kidney disease, the mean percentages were 24.38% for the sarcopenic and 17.65% for the non-sarcopenic group.

### 3.3. Primary and Secondary Outcomes

#### 3.3.1. Early Mortality and Late Mortality

**Figure 2:**
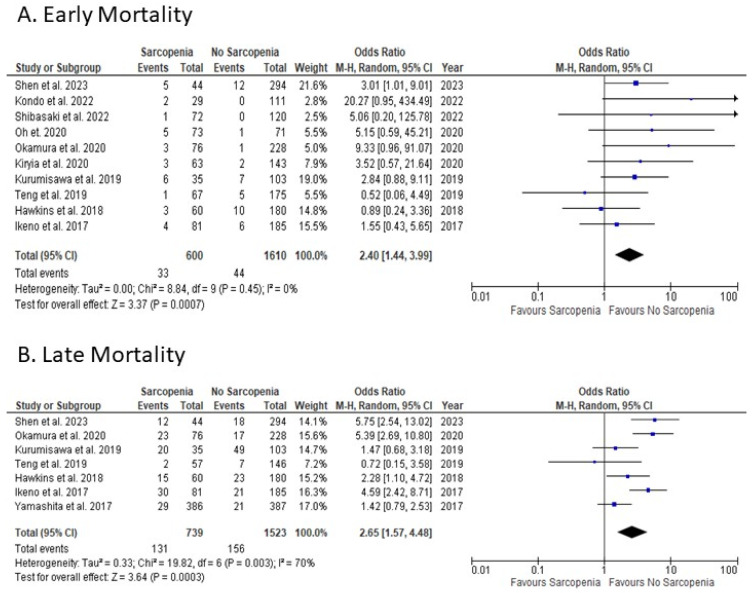
Forest Plots for the primary outcomes. (**A**) Early Mortality; (**B**) Late Mortality. References [12,13,14,17,18,19,20,21,22,23].

Sarcopenic patients were compared with non-sarcopenic patients, with ten studies reporting on early mortality outcomes postoperatively (Figure 2A). The overall OR for early mortality showed a statistically significant difference favouring non-sarcopenic patients (random-effects model: OR: 2.40; 95% CI: 1.44 to 3.99; *p* = 0.0007). There was evidence of no heterogeneity among studies reporting on early mortality.

Sarcopenic patients were compared with non-sarcopenic patients, with seven studies reporting on late mortality outcomes postoperatively (Figure 2B). The overall OR for late mortality showed a statistically significant difference favouring non-sarcopenic patients (random-effects model: OR: 2.65; 95% CI: 1.57 to 4.48; *p* = 0.0003). There was evidence of high heterogeneity among studies reporting on late mortality.

#### 3.3.2. Overall Surgery Time

**Figure 3:**
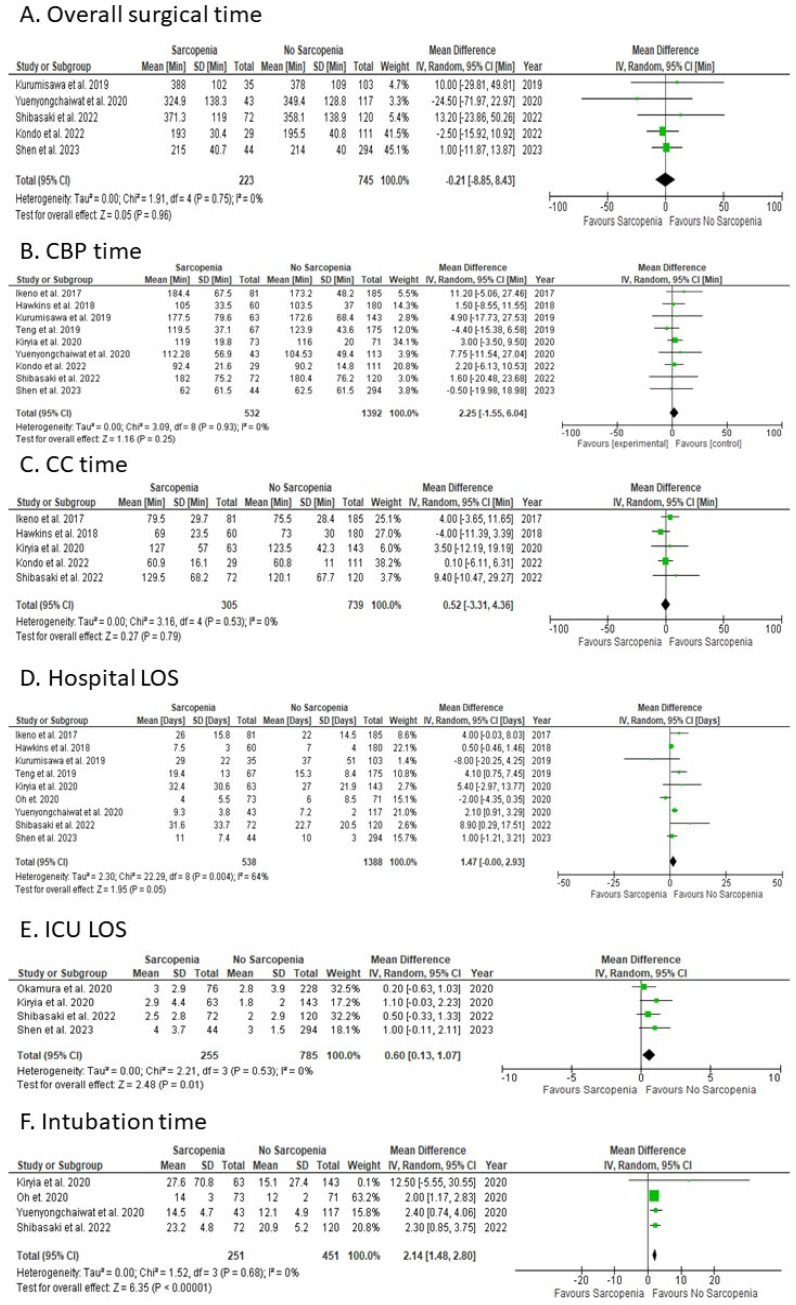
Forest Plots for the secondary outcomes focused on timing. (**A**) Overall surgical time; (**B**) CBP time; (**C**) CC time; (**D**) Hospital LOS; (**E**) ICU LOS; (**F**) Intubation time. References [12,13,14,15,16,17,18,20,21,22,23].

Sarcopenic patients were compared with non-sarcopenic patients, with five studies reporting overall surgery time (Figure 3A). The overall MD for overall surgery time showed no statistically significant difference between the two groups (random-effects model: MD: −0.21; 95% CI: −8.85 to 8.43; *p* = 0.96). There was evidence of no heterogeneity among studies reporting on overall surgical time.

#### 3.3.3. CBP Time

Sarcopenic patients were compared with non-sarcopenic patients, with nine studies reporting on CBP time (Figure 3B). The overall MD for CBP time showed no statistically significant difference between the two groups (random-effects model: MD: 2.25; 95% CI: −1.55 to 6.04; *p* = 0.25). There was evidence of no heterogeneity among studies reporting on CBP time.

#### 3.3.4. CC Time

Sarcopenic patients were compared with non-sarcopenic patients, with five studies reporting on CC time (Figure 3C). The overall MD for CC time showed no statistically significant difference (random-effects model: MD: 0.52; 95% CI: −3.31 to 4.36; *p* = 0.79). There was evidence of no heterogeneity among studies reporting on CC time.

#### 3.3.5. Hospital LOS

Sarcopenic patients were compared with no-sarcopenic patients, with nine studies reporting on Hospital LOS (Figure 3D). The overall MD for Hospital LOS showed no statistically significant difference (random-effects model: MD: 1.47; 95% CI: 0.00 to 2.93; *p* = 0.05). There was evidence of high heterogeneity among studies reporting on Hospital LOS.

#### 3.3.6. ICU LOS

Sarcopenic patients were compared with non-sarcopenic patients, with four studies reporting on ICU LOS (Figure 3E). The overall MD for ICU LOS showed a statistically significant difference favouring non-sarcopenic patients (random-effects model: MD: 0.60; 95% CI: 0.13 to 1.07; *p* = 0.01). There was evidence of no heterogeneity among studies reporting on ICU LOS.

#### 3.3.7. Intubation Time

Sarcopenic patients were compared with non-sarcopenic patients, with four studies reporting on intubation time (Figure 3F). The overall MD for intubation time showed a statistically significant difference favouring non-sarcopenic patients (random-effects model: MD: 2.14; 95% CI: 1.48 to 2.80; *p* < 0.0001). There was evidence of no heterogeneity among studies reporting on intubation time.

#### 3.3.8. Postoperative Arrhythmia

**Figure 4:**
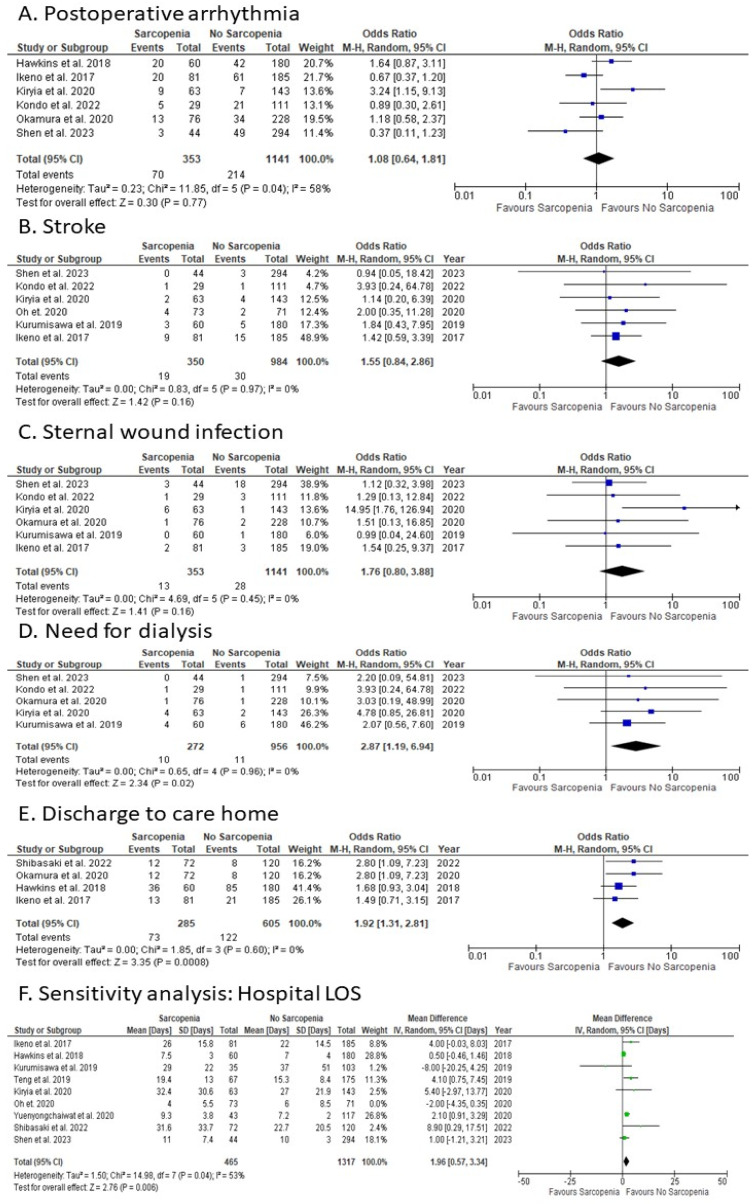
Forest Plots for the secondary outcomes. (**A**) Postoperative arrhythmia; (**B**) Stroke; (**C**) Sternal wound infection; (**D**) Need for dialysis; (**E**) discharge to care home; (**F**) Sensitivity analysis: Hospital LOS. References [12,13,14,15,16,17,18,20,21,22,23].

Sarcopenic patients were compared with non-sarcopenic patients, with six studies reporting on postoperative arrhythmia (Figure 4A). The overall OR for postoperative arrhythmia showed no statistically significant difference (random-effects model: OR: 1.08; 95% CI: 0.64 to 1.81; *p* = 0.77). There was evidence of moderate heterogeneity among studies reporting on postoperative arrhythmia.

#### 3.3.9. Stroke

Sarcopenic patients were compared with non-sarcopenic patients, with six studies reporting on stroke (Figure 4B). The overall OR for stroke showed no statistically significant difference (random-effects model: OR: 1.55; 95% CI: 0.84 to 2.86; *p* = 0.16). There was evidence of no heterogeneity among studies reporting on stroke.

#### 3.3.10. Sternal Wound Infection

Sarcopenic patients were compared with non-sarcopenic patients, with six studies reporting on sternal wound infection (Figure 4C). The overall OR for sternal infection showed no statistically significant difference (random-effects model: OR: 1.76; 95% CI: 0.80 to 3.88; *p* = 0.16). There was evidence of no heterogeneity among studies reporting on sternal infection.

#### 3.3.11. Postoperative Need for Dialysis

Sarcopenic patients were compared with non-sarcopenic patients, with five studies reporting on dialysis (Figure 4D). The overall OR for dialysis showed a statistically significant difference favouring non-sarcopenic patients (random-effects model: OR: 2.87; 95% CI: 1.19 to 6.94; *p* = 0.02). There was evidence of no heterogeneity among studies reporting on dialysis.

#### 3.3.12. Discharge to Care Home

Sarcopenic patients were compared with non-sarcopenic patients, with six studies reporting on care home discharge (Figure 4E). The overall OR for care home discharge showed a statistically significant difference favouring non-sarcopenic patients (random-effects model: OR: 1.92; 95% CI: 1.31 to 2.81; *p* < 0.001). There was evidence of no heterogeneity among studies reporting on care home discharge.

### 3.4. Sensitivity Analysis: Hospital LOS

Sensitivity analysis was carried out for all outcomes, with all outcomes other than Hospital LOS showing no statistically different impact on heterogeneity. Sarcopenic patients were compared with non-sarcopenic patients, with eight studies reporting on hospital LOS (Figure 4F). The overall MD for Hospital LOS showed a statistically significant difference favouring non-sarcopenic patients when the study by Oh et al. was removed (random-effects model: MD: 1.96; 95% CI: 0.57 to 3.34; *p* = 0.005). There was evidence of moderate heterogeneity among studies reporting on Hospital LOS.

### 3.5. Risk of Bias across the Studies

The funnel plot analysis (Supplementary Figures S1–S9) disclosed no asymmetry around the axis for the outcomes, thus making publication bias related to all outcomes unlikely.

## 4. Discussion

### 4.1. Sarcopenia and Mortality

The findings of our study demonstrate a significant difference in both early and late mortality between sarcopenic and non-sarcopenic patients undergoing cardiac surgery. These results are in line with the existing body of literature on the subject. Specifically, the OR for early mortality was 2.40 (95% CI: 1.44 to 3.99; *p* < 0.001), and for late mortality, it was 2.65 (95% CI: 1.57 to 4.48; *p* < 0.001). These results indicate that sarcopenic patients faced more than twice the risk of experiencing early mortality postoperatively compared to their non-sarcopenic counterparts. This elevated risk is likely attributed to the diminished physical reserve and heightened vulnerability to stressors, such as surgery, observed in sarcopenic patients [24].

Our findings align with the study by Englesbe et al., 2010 [25], which found sarcopenia to be a significant predictor of mortality in patients undergoing major elective general abdominal surgery and transplantation. The study reported an OR of 2.86, indicating that sarcopenic patients were nearly three times more likely to die than non-sarcopenic patients. Similarly, a systematic review and meta-analysis by Malietzis et al., 2016 [26] found that sarcopenia was associated with increased postoperative complications and mortality in patients undergoing gastrointestinal surgery.

Furthermore, the results of our study build upon the findings of Wayda et al., 2018 [27], supporting the independent association between socioeconomic status, as assessed by the Distressed Communities Index (DCI), and operative mortality following coronary artery bypass grafting (CABG). Wayda et al.’s study revealed that patients with low socioeconomic status (SES) based on the DCI had a 1.6-fold higher risk of postoperative mortality following CABG compared to patients with high SES. These findings suggest that socioeconomic factors, which might hypothetically influence the prevalence and impact of sarcopenia [28], also substantially influence patient outcomes.

The combined impact of sarcopenia on the overall mortality risk in surgical patients is noteworthy. Sarcopenia, often associated with aging, malnutrition, and physical inactivity, contributes to reduced muscle strength and poor physical performance [29], thereby elevating the risk of unfavourable postoperative outcomes. The presence of sarcopenia can exacerbate the challenges encountered by patients with low SES, who may already be at a higher risk of poor outcomes due to limited access to healthcare, poor nutrition, and higher stress levels.

### 4.2. Surgical Time

Our data showed no statistically significant difference in overall surgery time, CBP time, and CC time between sarcopenic and non-sarcopenic patients. This suggests that sarcopenia does not significantly prolong the duration of cardiac surgery. This is consistent with the findings of a study by Fukuda et al., 2016 [30], which found no significant difference in operation times between sarcopenic and non-sarcopenic patients undergoing gastric cancer surgery.

With the exception of patients with sarcopenic obesity, surgical access is not influenced by sarcopenia, and the technical aspects of the procedure can be carried out seamlessly, especially when one does not encounter significant amounts of pre-pericardial or epicardial fat that is often seen in obese patients. However, it is worth noting that the lack of significant difference in surgical time does not negate the potential impact of sarcopenia on other surgical outcomes.

### 4.3. Infection Rates

Our data showed no statistically significant difference in the rate of sternal infection between sarcopenic and non-sarcopenic patients. This is an interesting finding, as some studies have suggested that sarcopenia may increase the risk of postoperative infections. For instance, a study by Lieffers et al., 2012 [31] found that sarcopenia was associated with a higher risk of infection in cancer patients. It is recognised that obesity is the main risk factor for surgical site infections such as sternal wound infections [32,33]. Unless there is sarcopenic obesity or an active hypercatabolic state, sternal closure and healing should not be negatively influenced in sarcopenic patients, which would have led to significantly higher wound complications against the non-sarcopenic counterparts. This discrepancy may be due to differences in patient populations, surgical procedures, and infection prevention measures, warranting further investigation.

### 4.4. Functional Outcomes

Our data showed statistically significant differences in several functional outcomes between sarcopenic and non-sarcopenic patients. Sarcopenic patients had a longer ICU length of stay, higher likelihood of requiring dialysis, higher rate of care home discharge, and longer intubation time. These findings suggest that sarcopenia can significantly impact patients’ postoperative recovery and quality of life.

(a) ICU Length of Stay: The longer ICU stay for sarcopenic patients may be due to their lower physiological reserve and increased vulnerability to complications. Sarcopenia, characterised by a loss of muscle mass and function, can lead to frailty, which is associated with a higher risk of adverse outcomes, including prolonged ICU stay. This is supported by a study by Moisey et al., 2013 [34], which found that sarcopenic patients had a longer ICU stay after emergency abdominal surgery.
(b) Dialysis: The higher likelihood of requiring dialysis in sarcopenic patients probably relates to their increased risk of acute kidney injury (AKI) postoperatively. Sarcopenia may contribute to AKI through various mechanisms, including inflammatory pathway activation following chronic inflammation and increased susceptibility to nephrotoxic agents [35]. A study by Bang et al. [35] found that sarcopenia was an independent risk factor for AKI in patients undergoing abdominal aortic aneurysm surgery.
(c) Care Home discharge: The higher rate of care home discharge for sarcopenic patients may reflect their poorer functional status and increased need for assistance with daily activities postoperatively. Sarcopenia is associated with physical disability and reduced independence, which may necessitate care home admission. This is consistent with a study by Landi et al., 2012 [36], which found that sarcopenic older adults were more likely to be institutionalised.
(d) Intubation Time: The longer intubation time for sarcopenic patients may be due to their increased risk of respiratory complications. Sarcopenia can impair respiratory muscle function, leading to reduced lung volumes and ineffective cough, which can prolong the need for mechanical ventilation. A study by Puthucheary et al., 2013 [37] found that ICU-acquired weakness, which is often associated with sarcopenia, was a predictor of prolonged mechanical ventilation.

### 4.5. Measurements of Sarcopenia in Clinical Practice

Sarcopenia is typically identified and measured using a combination of methods that assess muscle mass, muscle strength, and physical performance. The EWGSOP and the AWGS have provided guidelines for the diagnosis of sarcopenia, which include the use of these three parameters [5,6]. However, in the practical setting of cardiac surgery, where patients often undergo preoperative CT scans, these existing images can be utilised to assess sarcopenia, making it a practical and cost-effective approach. The most common method of assessing sarcopenia using CT scans is by measuring the cross-sectional skeletal muscle area (SMA, cm^2^) at the level of the third lumbar vertebra, which is highly correlated with total body muscle mass. Adjusting the SMA for height squared yields the Skeletal Muscle Index (SMI, cm^2^/m^2^), a metric used to assess relative muscle mass. While the specific thresholds can vary, depending on age, body mass index (BMI), and definitions of sarcopenia, a commonly used threshold is an SMI less than 50–55 cm²/m² for men and less than 35–40 cm²/m² for women [38]. van der Werf et al. demonstrated a predicted 5th percentile SMI value (for all BMIs) of 36.9 cm^2^/m^2^ for men and 28.2 cm^2^/m^2^ for women in the age group of 70–79 years, indicating the extent and frequency of muscle mass loss in this age group [39]. Lastly, the psoas muscle has also been of particular interest as it can be easily visualised and measured on routine preoperative CT scans. The cross-sectional area of the psoas muscle has been used as a surrogate marker for total body muscle mass.

### 4.6. Sarcopenia and Frailty

The interaction between sarcopenia and frailty is complex and multifaceted, and both conditions often coexist in older adults, making their assessment and management challenging. Sarcopenia, characterised by the loss of muscle mass and strength, and frailty, a state of increased vulnerability to stressors due to decreased physiological reserves, are both prevalent conditions in older adults [24]. A cross-sectional study conducted by Gingrich et al. (2019) found that 42% of older medical inpatients had sarcopenia and 33% were frail, with these conditions overlapping in 19% of patients [40]. This indicates a significant interaction between the two syndromes, suggesting they may share common etiological factors such as reduced food intake, inflammation, hormonal changes, increased energy requirements, and reduced physical activity.

While sarcopenia can be measured relatively directly through assessments of muscle mass and strength, frailty, due to its multifaceted nature, is more challenging to measure. Frailty encompasses a decline in function across multiple organ systems, leading to increased vulnerability to stressors [41]. Various frailty indices and scales have been developed, such as the Fried Frailty Index and the Frailty Phenotype, but these require comprehensive clinical assessments and may not be feasible in all settings [41]. Furthermore, there is no universally accepted definition or measurement for frailty, leading to variability in how it is assessed and interpreted in clinical and research settings.

### 4.7. Recommendations for Optimising Cardiac Surgery Outcomes in Sarcopenic Patients

1. Preoperative identification of sarcopenia: Early identification of sarcopenia can allow for preoperative interventions to improve patient outcomes. This can be achieved through simple screening tools or more comprehensive assessments such as CT or MRI scans.
2. Preoperative optimisation: Once sarcopenia is identified, preoperative optimisation strategies should be implemented. This could include nutritional supplementation, physical therapy, and exercise programs aimed at increasing muscle mass and strength.
3. Risk stratification: Sarcopenic patients should be considered high-risk surgical candidates. This should be considered when planning the surgical approach and postoperative care.
4. Intraoperative care: Consideration should be given to minimizing operative time and blood loss, as sarcopenic patients may be more susceptible to intraoperative complications.
5. Postoperative rehabilitation: Early mobilisation and physical therapy should be initiated postoperatively to prevent further muscle loss and to promote recovery.
6. Nutritional support: Postoperative nutritional support should be provided to meet the increased protein and calorie needs of sarcopenic patients and to support muscle recovery.
7. Multidisciplinary approach: The care of sarcopenic patients should involve a multidisciplinary team, including surgeons, anaesthesiologists, dietitians, physical therapists, and geriatricians. This can ensure a comprehensive approach to the management of sarcopenia and its associated risks.
8. Patient education: Patients should be educated about the implications of sarcopenia and the steps they can take to improve their muscle health. This can empower patients to take an active role in their care and recovery.
9. Research: Further research should be conducted to better understand the impact of sarcopenia on cardiac surgery outcomes and to develop effective interventions for this patient population.

## 5. Conclusions

In conclusion, this meta-analysis highlights the significant adverse impact of sarcopenia on patients undergoing cardiac surgery. Sarcopenic patients demonstrate higher early and late mortality rates, longer ICU stays, increased likelihood of requiring dialysis, higher rates of care home discharge, and longer intubation times. These findings underscore the importance of recognizing sarcopenia as a significant risk factor in cardiac surgery.

Our findings align with existing literature across various surgical fields, further emphasizing the universal relevance of sarcopenia in surgical outcomes. Despite the heterogeneity in some of the studies, the overall trend suggests a consistently negative impact of sarcopenia on postoperative outcomes. However, our study also revealed that sarcopenia did not significantly affect certain outcomes, such as surgical time and infection rates. This suggests that the influence of sarcopenia may be more pronounced in certain areas, and its impact may be modulated by other factors such as surgical technique, perioperative care, and the patient’s overall health status. To optimize outcomes in sarcopenic patients, a comprehensive and multidisciplinary approach is recommended. This includes early identification and preoperative optimisation of sarcopenic patients, risk stratification, careful intraoperative management, postoperative rehabilitation, nutritional support, and patient education. Further research is needed to better understand the mechanisms underlying the impact of sarcopenia on surgical outcomes and to develop effective interventions.

## Figures and Tables

**Table 1:** Baseline characteristics of the included studies for both sarcopenic (SP) and non-sarcopenic (NSP) cohorts. Study designs included prospective (P) or retrospective (R) studies. Studies defined sarcopenia according to psoas muscle index (PMI), skeletal muscle index (SMI) on CT scan, Asian Working Group for Sarcopenia (AWGS), or European Working Group on Sarcopenia in Older People (EWGSOP). Not applicable (NA) has been used where studies do not report the specific parameter’s data. Left Ventricular Ejection Fraction (LVEF), Body Mass Index (BMI).

Study	Patient Numbers (SP.NSP)	Sarcopenia Definition	Study Design	Country	AGE				BMI				LVEF %				Psoas Muscle Area Index (mm^2^/m^2^)			
					Mean SP	SD SP	Mean NSP	SD NSP	Mean SP	SD SP	Mean NSP	SD NSP	Mean SP	SD SP	Mean NSP	SD NSP	Mean SP	SD SP	Mean NSP	SD NSP
Shibasaki et al., 2022	72/120	SMI: total body muscle	R	Japan	73.8	8.8	67.0	10.1	21.5	3.0	24.5	4.3	56.2	13.3	59.4	12.3	NA	NA	NA	NA
Kondo et al., 2022	29/111	PMI	R	Japan	81.0	5.8	77.3	4.7	21.6	4.2	22.8	3.7	62.2	9.0	62.1	9.0	704.0	112.0	1083.0	290.0
Okamura et al., 2020	76/228	PMI	P	Japan	69.9	8.9	66.6	9.7	21.6	3.0	24.1	3.3	55.5	14.4	56.4	13.9	168.0	37.0	283.0	65.0
Yuenyongchaiwat et al., 2020	43/117	AWGS	R	Thailand	66.4	10.7	59.1	11.2	NA	NA	NA	NA	53.3	15.1	51.9	13.7	NA	NA	NA	NA
Oh et al., 2020	73/71	SMI: pec. major & erector spinae	R	South Korea	59.5	13.1	60.4	10.6	22.6	3.2	24.4	3.0	60.0	3.0	60.0	3.0	NA	NA	NA	NA
Kurumisawa et al., 2019	35/103	PMI	R	Japan	67	10	66	9	19.5	2.9	22.6	3.8	50	15	52	16	NA	NA	NA	NA
Hawkins et al., 2018	60/180	PMI	R	USA	81	2	80	2.5	NA	NA	NA	NA	57	3	57	2.5	670.0	220.0	1060.0	280.0
Yamashita et al., 2017	387/386	PMI	R	Japan	65.5	13.2	64.4	13.1	23.4	3.53	21.1	3.45	57.8	12.5	56.7	13.1	401	78	667	111
Ikeno et al., 2017	81/185	PMI	R	Japan	76.2	5.6	45.7	5.7	22.5	3.4	23.8	2.9	NA	NA	NA	NA	NA	NA	NA	NA
Teng et al., 2019	67/175	EWGSOP	R	Japan	66.4	12.4	58.9	11.2	21.7	2.7	26.2	3.5	64.1	11.8	62.3	13.1	NA	NA	NA	NA
Shen et al., 2023	44/294	SMI: T12 level muscle	P	China	65.0	8.2	65.0	7.4	25.0	2.9	24.4	3.1	NA	NA	NA	NA	NA	NA	NA	NA
Kiriya et al., 2020	63/143	PMI	R	Japan	75.1	5.5	73.9	5.5	22.7	3.4	23.4	3.7	57.1	13.6	60.8	34.9	448.0	143.5	1005.0	312.5

References: [12,13,14,15,16,17,18,19,20,21,22,23].

## Data Availability

All the data is available upon request from the corresponding author (A.K.).

## Supplementary Materials

The following supporting information can be downloaded at: https://www.mdpi.com/article/10.3390/jcm12175573/s1, Figure S1: Overall Surgery Duration; Figure S2: CBP Time; Figure S3: CC Time; Figure S4: Hospital LOS; Figure S5: ICU LOS; Figure S6: In hospital mortality; Figure S7: Early mortality; Figure S8: Late mortality; Figure S9: Arrhythmia; Table S1: Continuous baseline characteristics of the included studies.
