# Supplementary material for: Sarcopenia Adversely Affects Outcomes following Cardiac Surgery: A Systematic Review and Meta-Analysis
Source: J Clin Med. 2023 Aug 26;12(17):5573. doi: 10.3390/jcm12175573 (PMC10488406; doi:10.3390/jcm12175573)
Supplement: Supplementary file 1 [file jcm-12-05573-s001.zip › jcm-2555189-supplementary.pdf]

Supplementary File 1

Supplementary Figure S1: Overall Surgery Duration.

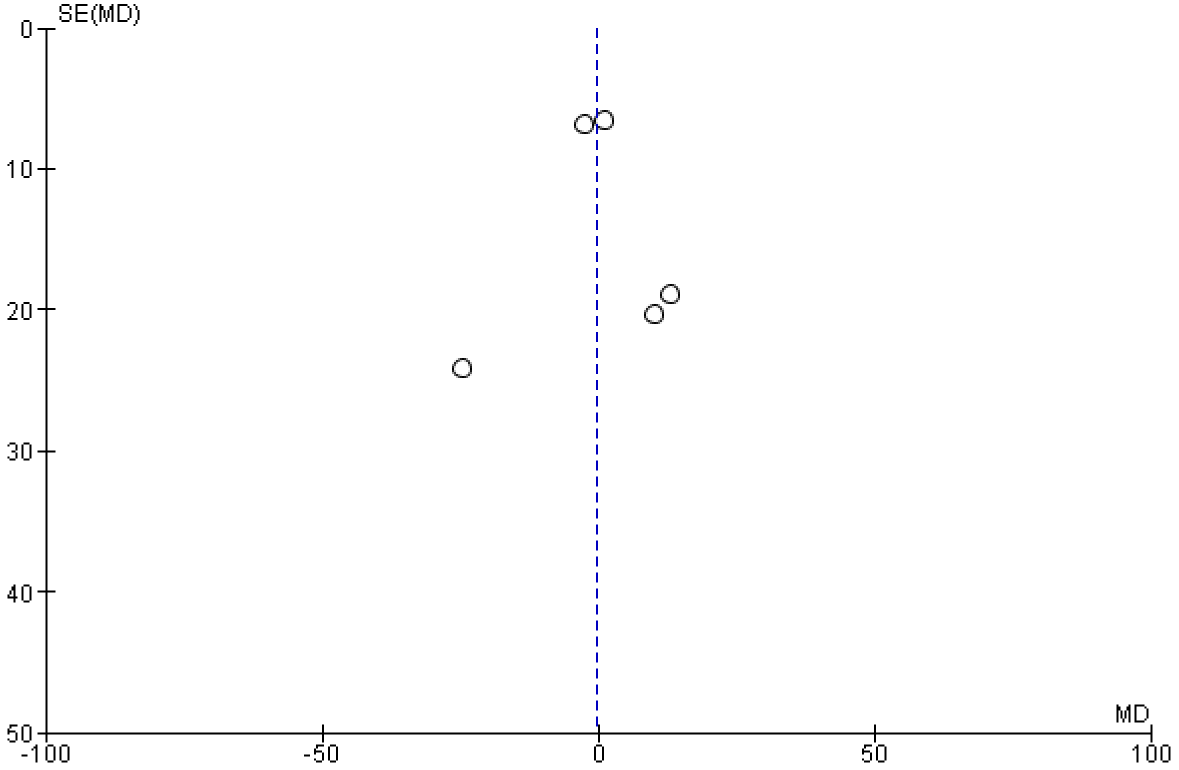

Supplementary Figure S2: CBP Time.

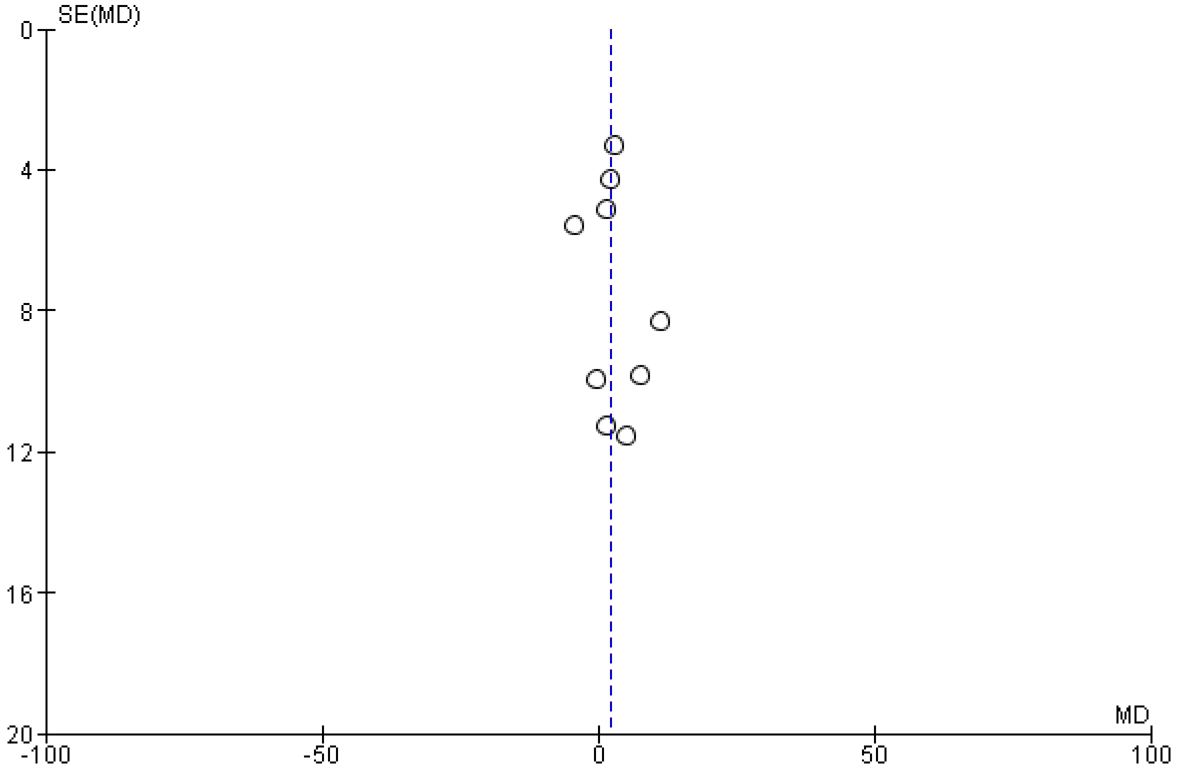

Supplementary Figure S3: CC Time.

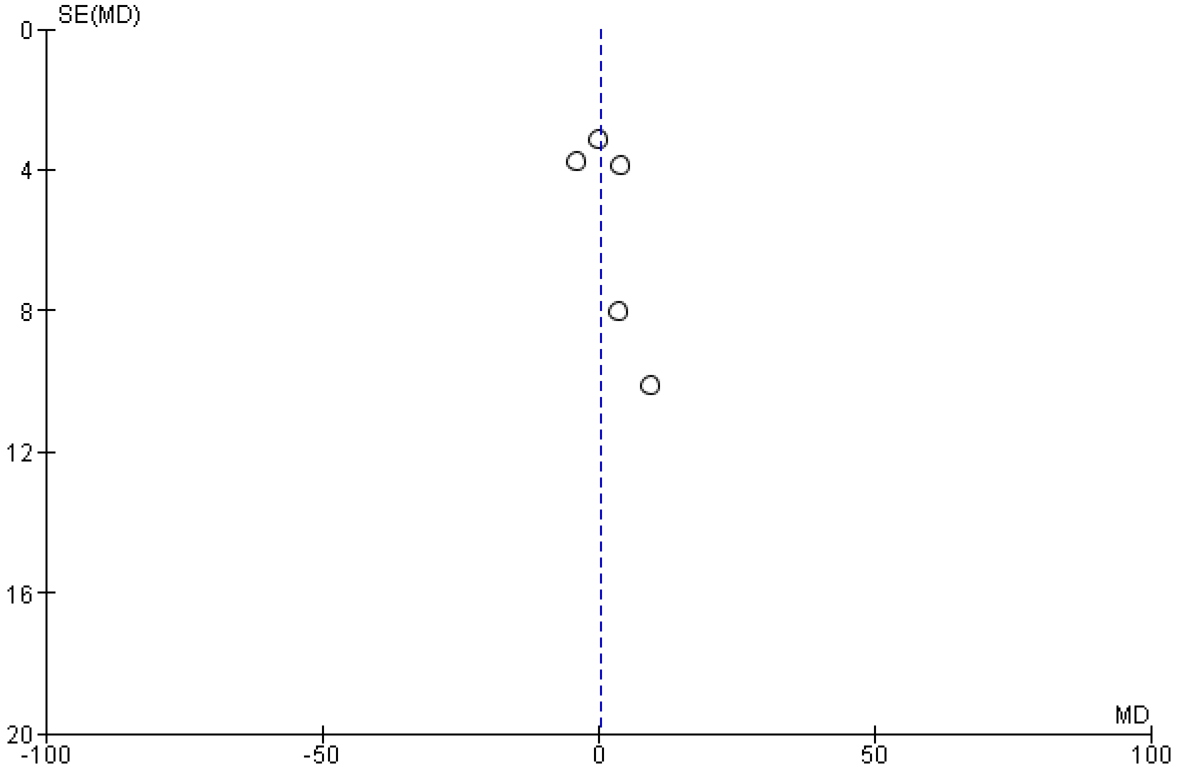

Supplementary Figure S4: Hospital LOS.

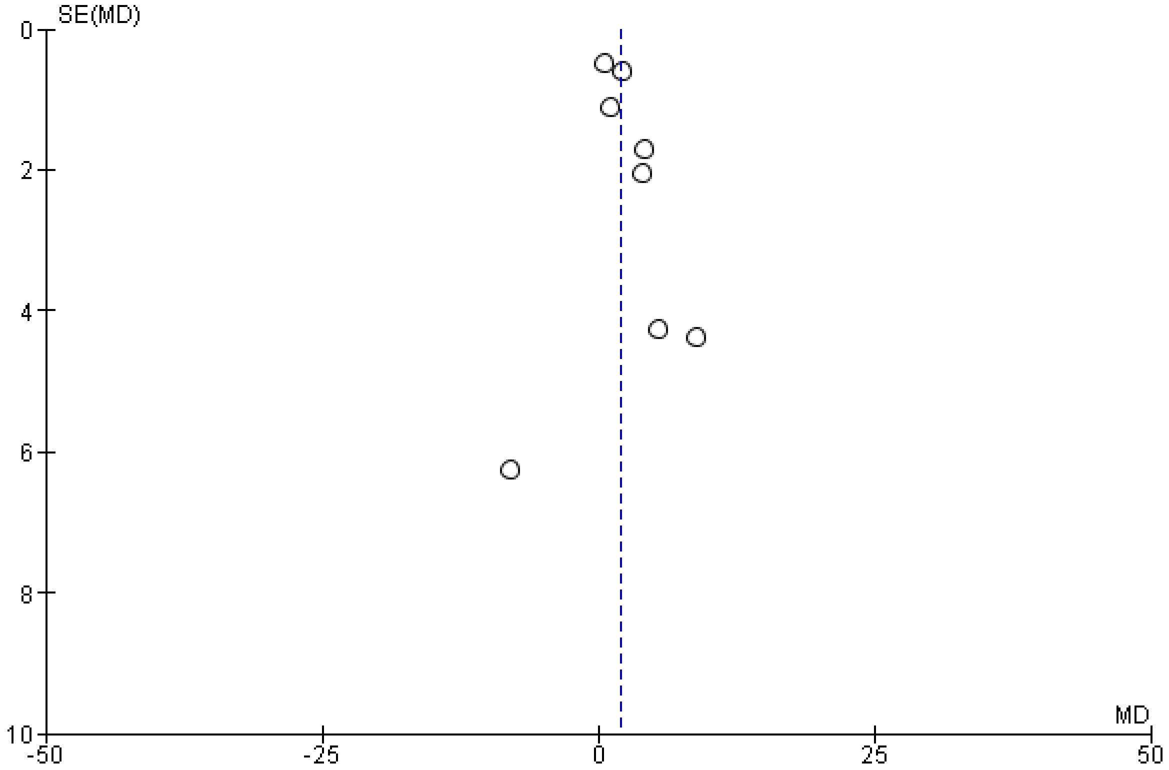

Supplementary Figure S5: ICU LOS.

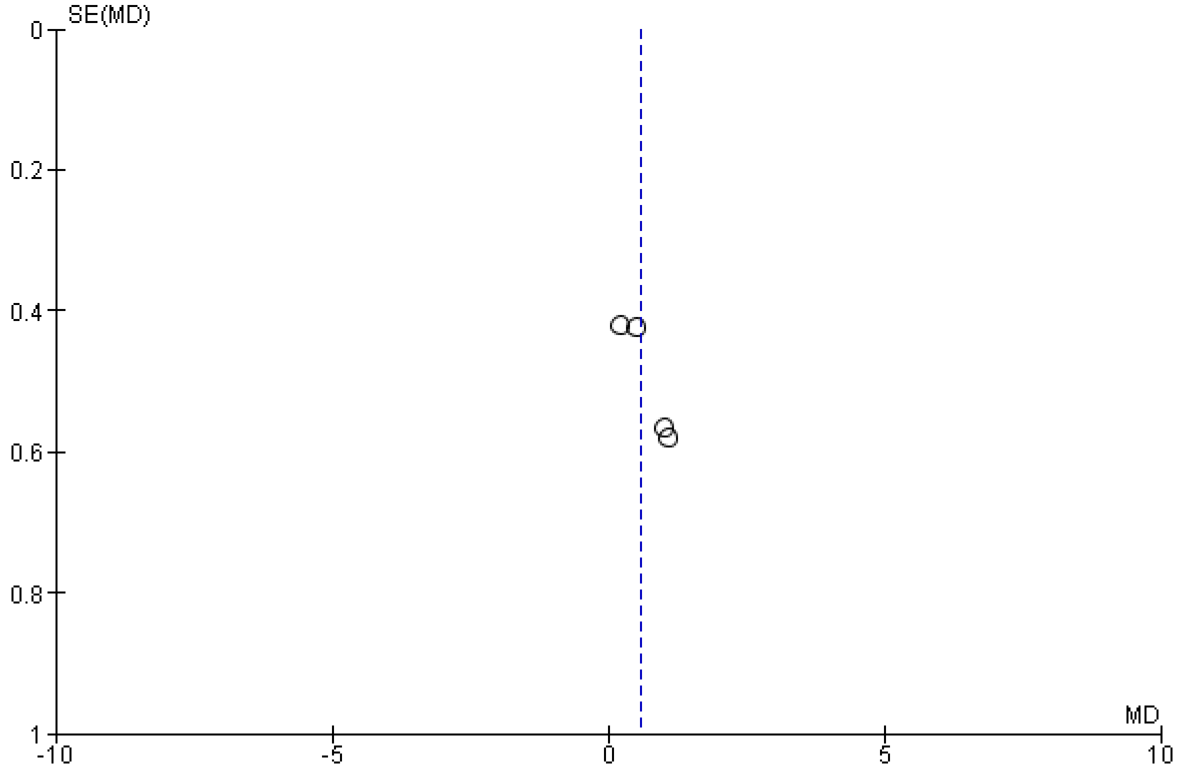

Supplementary Figure S6: In hospital mortality.

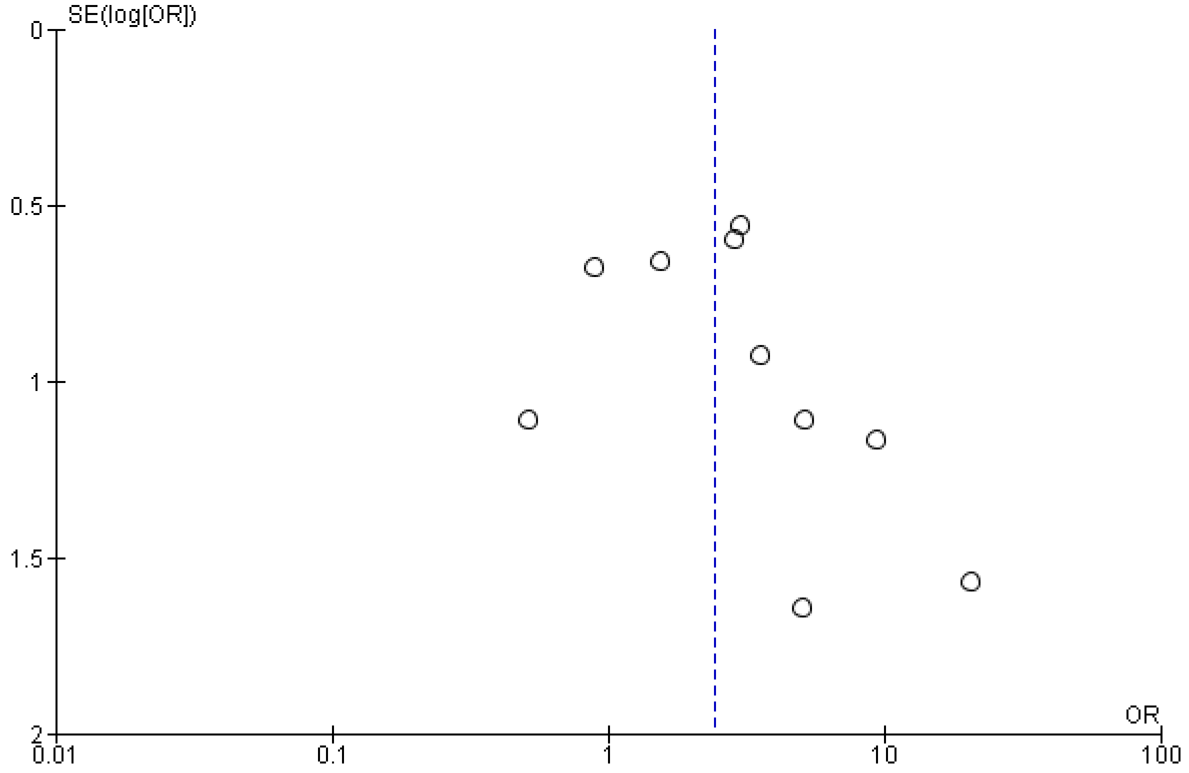

Supplementary Figure S7: Early mortality.

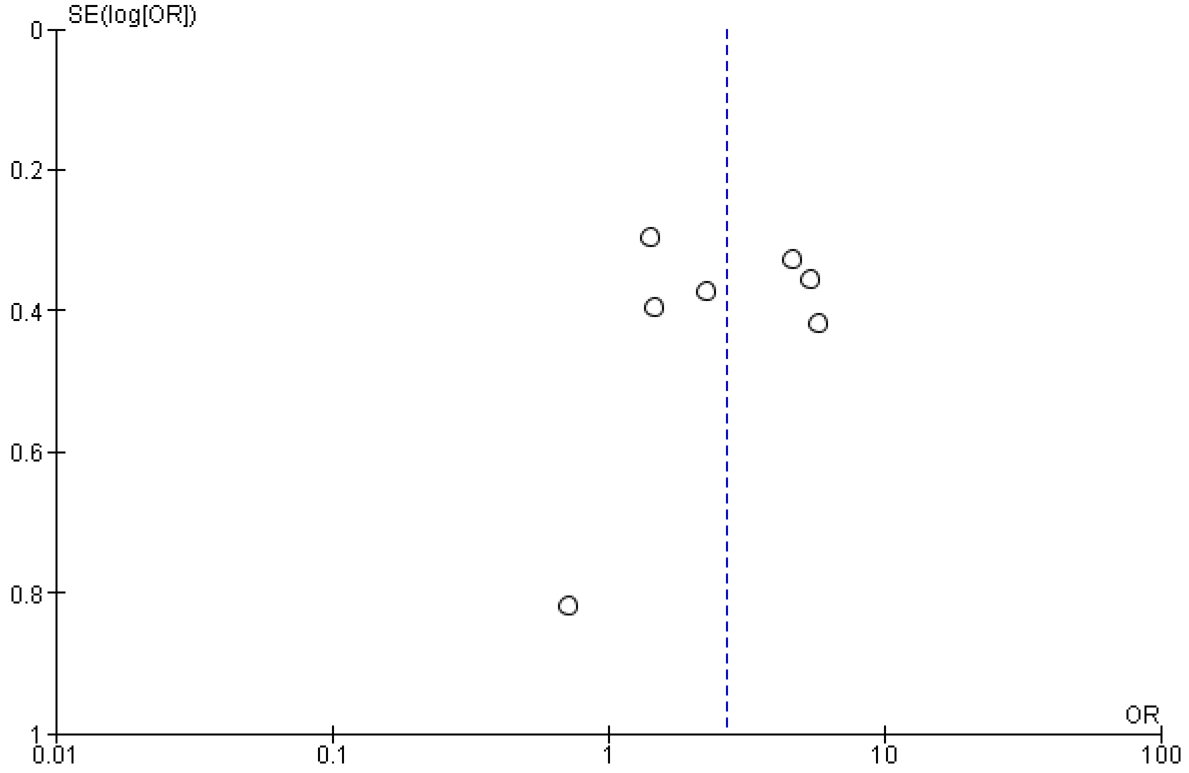

Supplementary Figure S8: Late mortality.

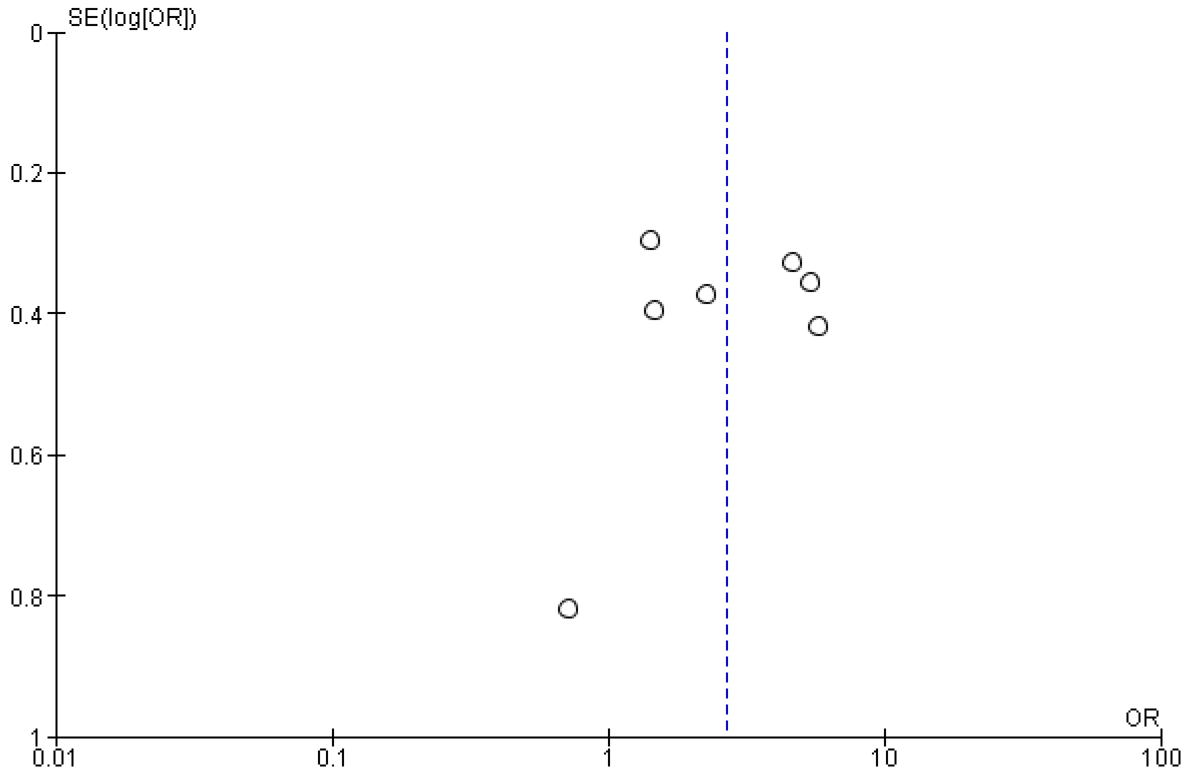

Supplementary Figure S9: Arrhythmia.

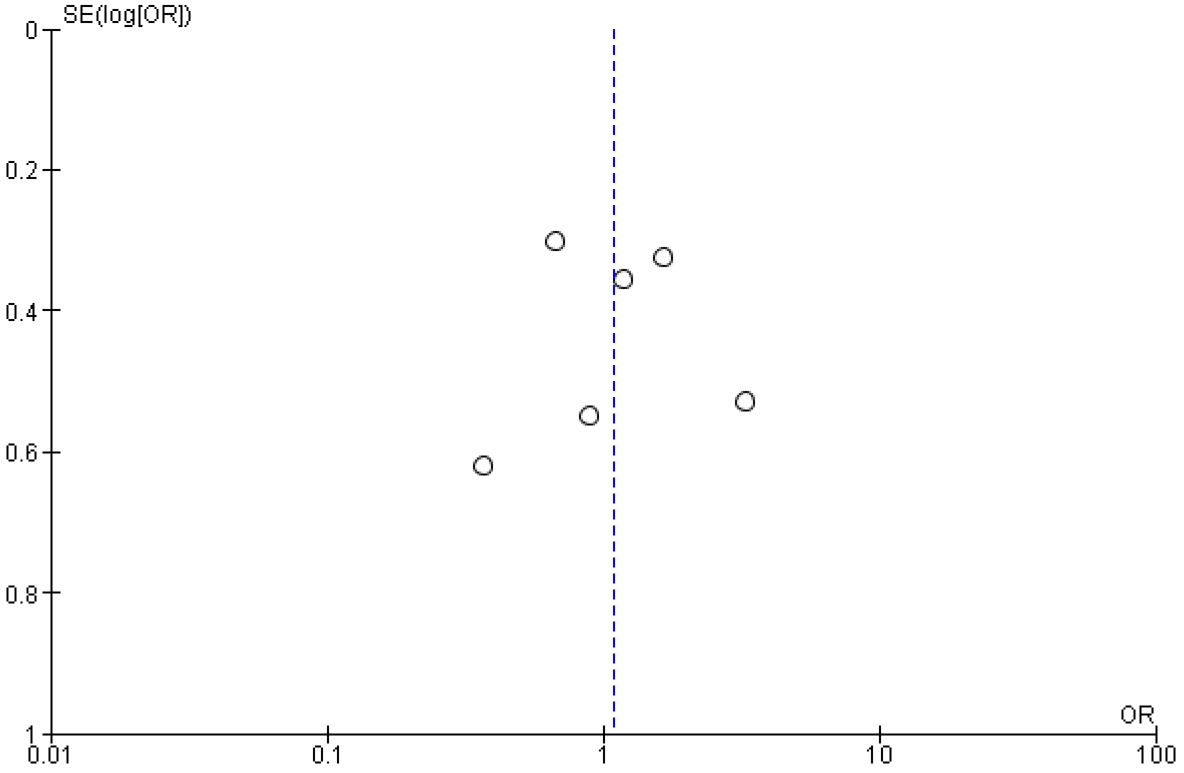

Supplementary Table S1: Continuous baseline characteristics of the included studies.

Study	**Diabetes Sarcopenic %**	**Diabetes Non-Sarcopenic %**	**HTN Sarcopenic %**	**HTN Non-Sarcopenic %**	**CKD Sarcopenic %**	**CKD Non-Sarcopenic %**	**CVD Sarcopenic %**	**CVD Non-Sarcopenic %**
Shen et al. 2023	50.0	48.98	75.0	80.61	4.54	5.44	15.90	14.96
Shibasaki et al. 2022	37.5	35.0	73.61	79.17	23.61	8.33	N/A	N/A
Kondo et al. 2022	24.14	22.52	51.72	67.57	10.34	14.41	3.44	3.60
Okamura et al. 2020	50.0	46.05	60.53	66.23	23.68	12.28	14.4	13.15
Yuenyongchaiwat et al. 2020	100.0	21.37	79.07	67.52	20.93	12.82	N/A	N/A
Oh et. 2020	N/A	N/A	N/A	N/A	N/A	N/A	N/A	N/A
Kiryia et al. 2020	38.10	38.46	84.13	74.13	N/A	N/A	N/A	N/A
Kurumisawa et al. 2019	N/A	N/A	74.29	67.96	N/A	N/A	28.57	19.41
Teng et al. 2019	23.88	30.29	55.22	54.86	20.89	9.71	N/A	N/A
Hawkins et al. 2018	43.33	45.56	85.0	90.0	N/A	N/A	15	10

Yamashita et al. 2017	29.27	37.47	56.74	67.44	N/A	N/A	N/A	N/A
Ikeno	19.75	18.38	79.01	89.73	66.66	60.54	N/A	N/A
